# Evaluation of Wearable Sensor Placement for Movement Bout Duration Estimation: A Comparative Feasibility Study

**DOI:** 10.3390/bioengineering13101206

**Published:** 2026-10-16

**Authors:** Serena Cerfoglio, Lucia Donno, Mattia Lovato, Carlalberto Francia, Manuela Galli, Veronica Cimolin

**Affiliations:** 1 Department of Electronics, Information and Bioengineering, Politecnico di Milano, Piazza Leonardo da Vinci 32, 20133 Milano, Italy; serena.cerfoglio@polimi.it (S.C.); lucia.donno@polimi.it (L.D.); mattia.lovato@polimi.it (M.L.); carlalberto.francia@polimi.it (C.F.); manuela.galli@polimi.it (M.G.); 2 IRCCS Istituto Auxologico Italiano, San Giuseppe Hospital, Strada Luigi Cadorna 90, 28824 Piancavallo, Italy; 3 E4Sport-Human Peformance Lab, Polo Territoriale di Lecco, Politecnico di Milano, Via G. Previati 1/c, 23900 Lecco, Italy

**Keywords:** wearable sensors, inertial measurement units, sensor placement, movement segmentation, temporal accuracy, free-living monitoring

## Abstract

Wearable sensor placement can influence movement quantification, yet direct comparisons across anatomical sites remain limited for bout-level temporal outcomes. This study evaluated seven IMU placements for movement bout duration estimation. Twenty-seven healthy adults performed three whole-body tasks (walking, Timed Up and Go, and Step Test) and two upper-limb tasks (reaching and hand-to-mouth) under concurrent optoelectronic motion capture. IMU-derived durations were compared with segment-specific local references and, for whole-body tasks, a common sacral reference used as a proxy for whole-body movement. Agreement was assessed using Spearman’s correlation, ICC, Bland–Altman analysis, and MAPE. Participant-level agreement analyses were complemented by trial-level repeated-measures analyses to account for within-participant variability across repeated trials. A descriptive composite ranking combined ICC, MAPE, and limits-of-agreement width against the common reference. Performance varied across placements and tasks, with the largest local–common dissociation observed during the TUG. The sacrum obtained the most favorable overall composite ranking score (2.78), closely followed by the dominant ankle (2.89) and non-dominant wrist (3.11). Both wrists showed high agreement during upper-limb tasks (ICC = 0.879–0.994). Trial-level analyses confirmed a significant effect of sensor placement for all whole-body tasks, while no significant differences were observed between wrist placements during the isolated upper-limb tasks. The non-dominant wrist showed a favorable combination of performance across movement domains, supporting its selection as a promising candidate for further evaluation in subsequent free-living monitoring.

## 1. Introduction

Wearable inertial sensors based on Inertial Measurement Units (IMUs) are widely used for the objective monitoring of human movement beyond controlled laboratory settings [1,2,3]. Among their applications, accelerometer-based monitoring enables physical activity to be recorded continuously over prolonged periods, providing information on movement behavior in everyday environments and complementing traditional laboratory-based instrumental assessments [4,5,6,7].

A key methodological aspect in wearable monitoring is sensor placement. The acceleration recorded during the same activity can vary substantially across anatomical sites, as different body segments move with different amplitudes and patterns [8,9]. Differences have been reported between wrist- and hip-worn recordings and between dominant and non-dominant wrists [10]. Distal lower-limb sensors are more directly related to stepping and locomotor events, trunk-mounted sensors better reflect global body movement, and wrist-mounted sensors also capture independent upper-limb activity [11,12,13]. Accordingly, sensor placement can affect derived outcomes such as movement quantification, activity recognition, and energy-expenditure estimation [14,15,16,17].

Accelerometer data can be processed to derive different measures of physical activity and movement. Common metrics such as vector magnitude, Euclidean Norm Minus One (ENMO), and Mean Amplitude Deviation (MAD) are used to quantify movement intensity or volume [18,19]. Beyond these summary measures, accelerometer signals can also be used to identify discrete periods of activity and quantify their temporal characteristics, including bout duration [20,21]. The accuracy of these outcomes depends on both the processing approach and the anatomical placement of the sensor, and should therefore be evaluated specifically for the intended application [22].

Existing validation studies have typically focused on a predefined sensor location. For example, the Mobilise-D consortium assessed gait-sequence and bout-level digital mobility outcomes using a single lower-back sensor against a laboratory reference system [23], while a recent systematic review similarly reported a predominance of single-location validation studies [24]. Multi-placement studies have more commonly addressed physical activity intensity, activity recognition, or energy expenditure [17,19]. Direct comparisons of several anatomical sites against the same reference therefore remain less common for bout-level temporal outcomes. This is relevant because a sensor may accurately track the segment on which it is worn without necessarily providing an equally accurate representation of the overall motor task.

Sensor placement also has practical implications for prolonged free-living monitoring. Although some anatomical sites provide higher measurement accuracy, they are not always suitable for continuous use because of comfort, acceptability, or interference with daily activities [25]. Therefore, the optimal placement may represent a compromise between technical performance and practical feasibility. This aspect is particularly relevant in populations requiring long-term wearable monitoring, where adherence to device use is essential and sensory factors may influence acceptance [26].

The present work focused on identifying a suitable sensor placement in healthy adults under controlled laboratory conditions. Rather than developing task-specific movement parameters, the study used active movement bout duration as a common temporal outcome, allowing different anatomical placements to be compared across a range of predefined motor tasks. The primary aim was to compare the performance of seven IMU placements for estimating active movement bout duration using concurrent optoelectronic motion capture as reference.

The secondary aims were to investigate whether placement performance varied according to the motor task and whether accurate tracking of the instrumented segment-specific duration estimates accurately represented the broader movement bout. The findings will provide a technical basis for selecting the sensor configuration and guiding the subsequent development of free-living monitoring.

## 2. Materials and Methods

### 2.1. Participants

The study was conducted at the “Luigi Divieti” Posture and Movement Analysis Laboratory of Politecnico di Milano (Milan, Italy) between October and December 2025. Twenty-seven healthy adults (14 females and 13 males; age: 26.33 ± 1.63 years; height: 171.81 ± 7.57 cm; weight: 65.15 ± 12.10 kg; BMI: 21.91 ± 2.91 kg/m^2^) were enrolled in the study on a voluntary basis. Participants were included if they had a BMI within the normal range (18.5–24.9 kg/m^2^) and no self-reported musculoskeletal conditions potentially affecting gait or upper-limb function. All participants were right-handed.

The study protocol was approved by the Ethics Committee of Politecnico di Milano (protocol n. 49/2025) and it was conducted in accordance with the ethical principles outlined in the Declaration of Helsinki (1964) and its subsequent amendments. All participants received detailed information about the study procedures and their right to withdraw at any time and provided written informed consent.

### 2.2. Experimental Setup and Testing Protocol

Optical data were collected using an eight-camera marker-based motion capture (MoCap) system (SMART DX 400, BTS Bioengineering S.p.A., Milan, Italy) with a sampling frequency of 100 Hz. Passive reflective markers were placed bilaterally on selected anatomical landmarks, including the wrists, heels, lateral malleoli, and metatarsal regions, with an additional marker positioned over the first sacral vertebra (S1).

**Figure 1:**
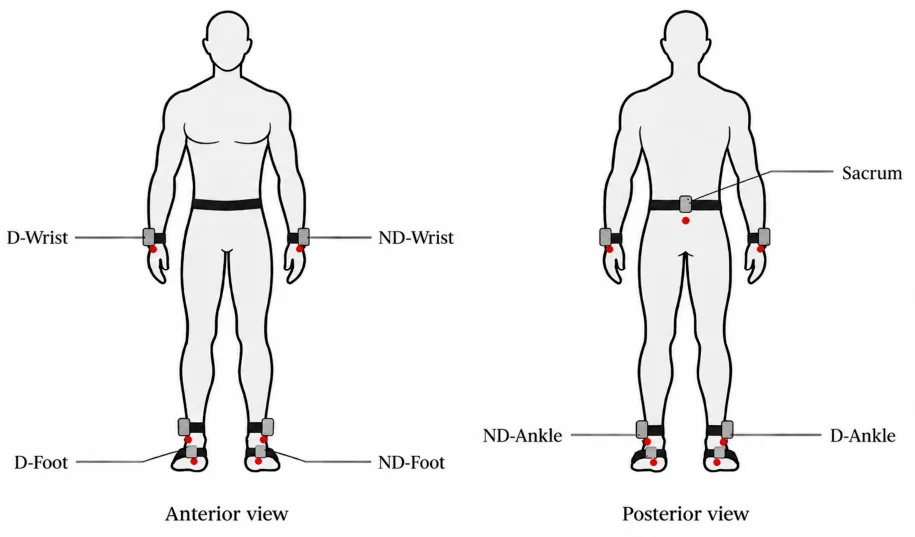
Placement of the IMUs in anterior (**left**) and posterior (**right**) views. D = dominant side, ND = non-dominant side, mirrored per participant. Red dots: retro-reflective markers tracked by the MoCap reference system.

Simultaneously, participants were equipped with seven IMUs (AX6, Axivity Ltd., Newcastle upon Tyne, UK), sampling at 100 Hz. The device is a compact six-axis inertial unit (23 × 32.5 × 8.9 mm; 11 g) with configurable accelerometer and gyroscope range. For the present study, the accelerometer was configured with a range of ±8 g, while the gyroscope was set to ±2000°/s. Although gyroscope signals were acquired during data collection, only accelerometer data were considered. The IMUs were positioned bilaterally on the wrists, ankles, and dorsum of the feet, with an additional sensor placed on the lower back over the sacral region. These locations were selected to include both distal and proximal body segments, as commonly adopted in wearable-based movement analysis [27]. All sensors were secured using elastic straps to minimize relative displacement between the device and the underlying body segment. The complete experimental setup is shown in Figure 1.

Before data collection, a static calibration recording was performed for approximately 20 min. During this procedure, sensors were placed on a horizontal surface with the z-axis oriented upward and remained stationary. The calibration recording was used to estimate sensor-specific acceleration bias relative to gravitational acceleration, according to the procedure described by Mazzà et al. [28].

**Figure 2:**
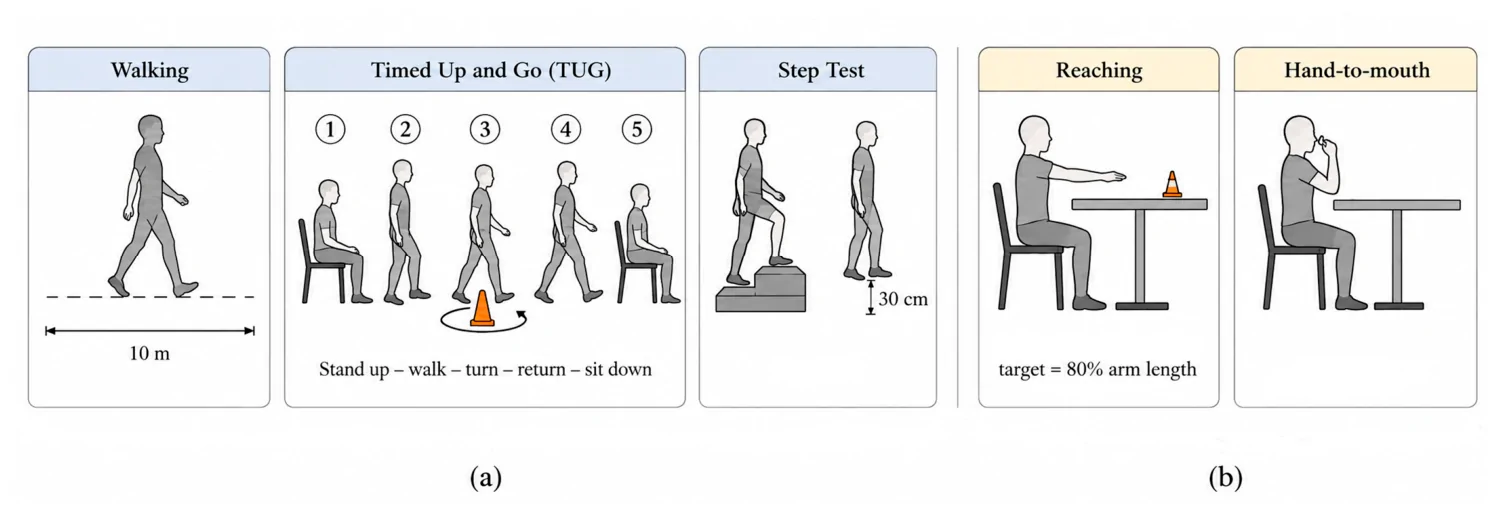
Schematic representation of the experimental motor tasks. (**a**) Whole-body tasks: 10 m walking, Timed Up and Go (TUG), and Step Test. (**b**) Upper-limb tasks: reaching and hand-to-mouth movements.

Participants performed a set of motor tasks selected to include both whole-body movements and upper-limb activities (Figure 2). As for whole-body movements, the following tasks were performed:

• Walking: starting from an upright standing position, nine trials of walking along a 10 m walkway were collected.
• Timed Up and Go (TUG): starting from a seated position, the standard TUG test [29]. Three trials were performed, each consisting of standing up from a chair, walking over a predefined distance, turning, returning to the chair, and sitting down.
• Step Test: starting from a standing position, three repetitions of the test were performed for each limb. During each trial, participants completed six consecutive ascent–descent cycles on a 30 cm step with the same limb, followed by six repetitions with the contralateral limb.

These tasks were selected to assess movement bout duration estimation across different movement patterns, including locomotion, postural transitions, and rhythmic lower-limb movements. Together, they provide a range of controlled conditions representative of movements commonly performed in daily life.

For specific upper-limb movements, two tasks were included:

• Reaching: from a seated position with both wrists resting on a table, participants reached and touched a target positioned at 80% of their arm length to standardize movement amplitude across participants. Six repetitions per side were performed for two series at a natural self-selected speed.
• Hand-to-mouth task: from the same seated starting position, participants brought the hand to the mouth and returned it to the initial position. Six repetitions per side were performed for two series at a natural self-selected speed.

The upper-limb tasks were included to assess bout duration estimation when movement is primarily expressed at the wrist, rather than as part of a whole-body activity.

### 2.3. Signal Processing and Active Bout Detection

The signal processing pipeline was designed in Matlab (R2025a, MathWorks, Natick, MA, USA) to identify active movement bouts from both MoCap and IMU recordings. MoCap-derived bout durations were used as reference values for the evaluation of IMU-based estimates. IMU and MoCap data were acquired concurrently during the same predefined experimental trials. No formal sample-level synchronization was performed, as the analysis focused on the duration of complete predefined movement sequences. Signals were independently processed and segmented within each trial, and the resulting bout durations were compared between systems.

#### 2.3.1. MoCap-Based Reference Bout Detection

MoCap marker trajectories were used to define the reference active movement bouts. Marker-based motion capture was selected as the reference system because it enables accurate three-dimensional tracking of anatomical landmarks and provides reliable estimates of spatiotemporal and kinematic gait parameters. Accordingly, MoCap is commonly adopted as the reference method in validation studies assessing the accuracy of IMU-based gait analysis systems [24].

Depending on the task and sensor placement, trajectories from the sacral marker (S1), bilateral heel markers, and bilateral wrist markers were considered. Short gaps in marker trajectories were linearly interpolated before filtering and segmentation. No major trajectory gaps requiring exclusion were observed, as marker tracking quality was adequate throughout the acquisitions. Marker trajectories were low-pass filtered using a fourth-order zero-lag Butterworth filter with an 8 Hz cut-off frequency [30]. The three-dimensional velocity magnitude was calculated from the filtered trajectories and smoothed using a 0.30 s moving average window. Velocity-based kinematic signals were used to identify active movement periods, as marker-derived velocity profiles have been widely used for movement segmentation [31,32].

Active intervals were identified using marker-specific adaptive hysteresis thresholds, automatically derived from the velocity signal distribution to account for differences in movement amplitude across anatomical locations and tasks. For low-amplitude signals (maximum velocity < 0.25 m/s), thresholds were estimated from baseline signal variability (mean plus 3 and 2 standard deviations of samples below the 20th percentile, for onset and offset, respectively), whereas for higher-amplitude signals, thresholds were calculated using the median and median absolute deviation (MAD) of the velocity signal (median plus 1.5 and 1.0 MAD, respectively). Threshold crossings were required to persist for at least 0.08 s (onset) and 0.15 s (offset). The same segmentation procedure was consistently applied across participants for each marker location and task.

Short gaps within detected intervals (<2 s) were merged to avoid fragmentation of continuous movement bouts, and intervals shorter than 1.5 s were discarded. In the present study, a movement bout was defined as the complete predefined movement sequence performed within each experimental trial rather than as an individual repetition. Accordingly, the gap-merging criterion was used to prevent short pauses between consecutive repetitions belonging to the same predefined task from artificially fragmenting a single movement bout. Detected bouts were visually checked to confirm correspondence with task execution.

The resulting MoCap-derived durations were used as references for comparison with IMU-derived estimates.

#### 2.3.2. IMU-Based Bout Detection

For the IMU data, tri-axial acceleration signals were processed following the static calibration performed before data collection, which was used to correct potential sensor offset. Acceleration signals were then low-pass filtered using a fourth-order zero-lag Butterworth filter with a 20 Hz cut-off frequency [33].

The Euclidean Norm Minus One (ENMO) was used as a summary metric of body-worn accelerometer signals. ENMO is a widely used metric for processing raw accelerometer data and has been applied for the quantification of human movement using body-worn sensors [18,19]. ENMO was calculated as follows:

(1) $$ENMO\left(t\right)=\max\left(\sqrt{{x\left(t\right)}^{2}+{y\left(t\right)}^{2}+{z\left(t\right)}^{2}}-1,\;0\right)$$

where x(t), y(t), and z(t) represent the tri-axial acceleration components in the time domain, expressed in gravitational units (g). The subtraction of g removes the static gravitational component, while negative values are truncated to zero.

ENMO signals were smoothed using a moving-average filter with a common 0.30 s window duration across all placements. Active bouts were identified using hysteresis thresholding with location-specific onset and offset thresholds. Thresholds were selected based on previously reported ENMO-based approaches and defined separately for each anatomical sensor location. This choice was motivated by the different acceleration amplitudes expected at distal (wrist and foot/ankle) and proximal (sacral) body segments during the same motor task, which may affect the sensitivity of a single common threshold across placements [19,34]. The applied onset/offset thresholds were 0.050/0.035 g for wrist sensors, 0.025/0.020 g for ankle and foot sensors, and 0.040/0.030 g for the sacral sensor. The same threshold pair was applied to both sides of the same anatomical location and was kept fixed across participants and trials.

Short gaps within detected intervals (<2 s) were merged to avoid fragmentation of continuous movement bouts, and intervals shorter than 1.5 s were discarded for all sensor placements, consistently with the MoCap-based reference processing. As for the MoCap segmentation, these criteria were intended to identify the complete predefined motor sequence within each controlled experimental trial rather than individual repetitions.

#### 2.3.3. Comparison of Bout Durations Between IMU and MoCap

IMU-derived bout durations were compared with MoCap-derived durations using two reference definitions, designed to distinguish segment-specific movement duration from a broader representation of the motor task.

The local reference was defined using the MoCap marker corresponding to the anatomical segment instrumented with the IMU, specifically the ipsilateral wrist marker for wrist sensors, heel markers for lower-limb sensors, and the S1 marker for the sacral sensor. This comparison assessed agreement between IMU-derived durations and the movement duration of the corresponding segment.

**Figure 3:**
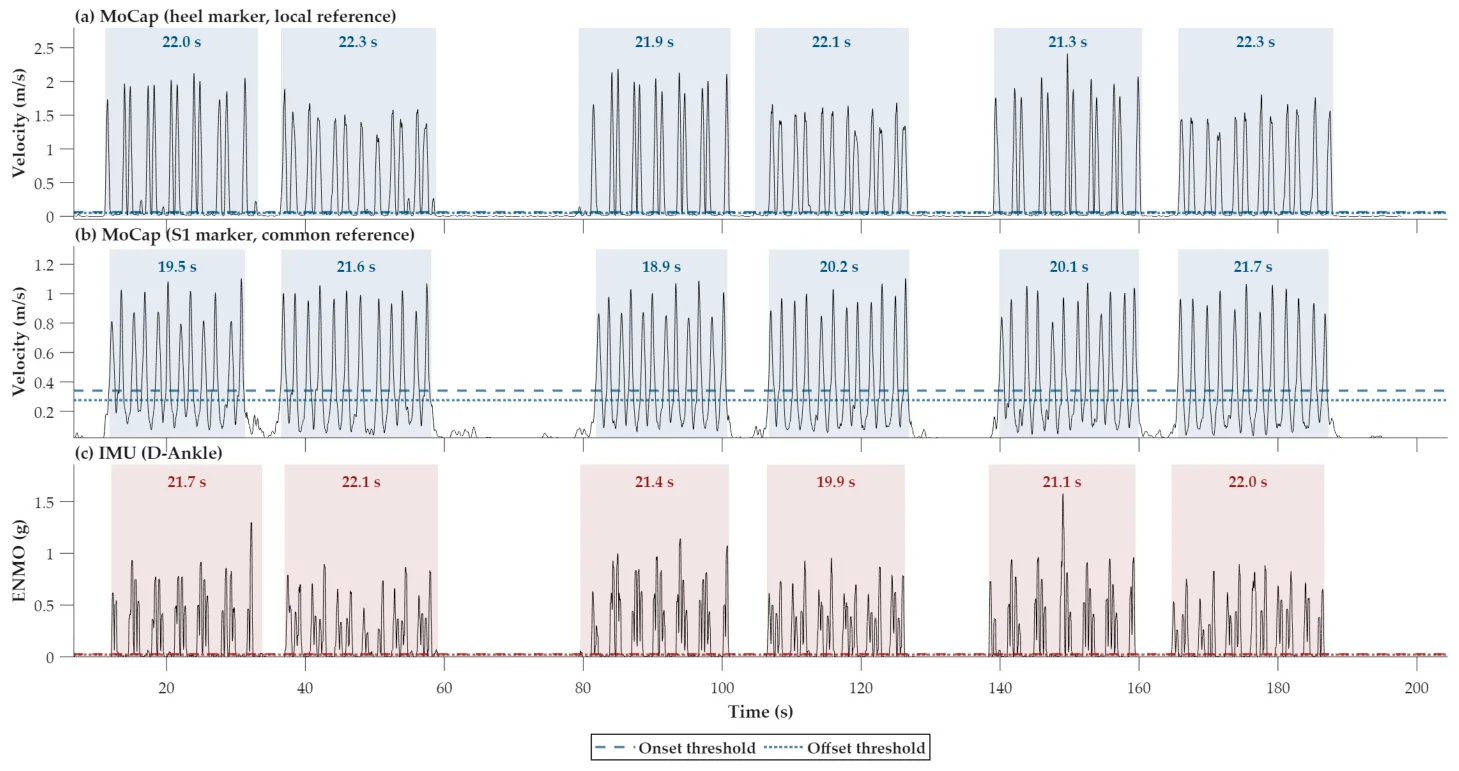
Active bout detection during the Step Test in a representative subject. From top to bottom: dominant heel marker (local reference), S1 marker (common reference), and IMU on the dominant ankle. Dashed and dotted lines indicate onset and offset thresholds; shaded areas indicate detected bouts.

The common reference was defined using the S1 marker as a proxy for whole-body movement. The sacral region was selected because of its proximity to the body’s center of mass (COM) and its established use in movement assessment [35]. This marker therefore provided a consistent kinematic reference against which all sensor placements could be compared, rather than an absolute measure of global body motion. This approach allowed us to assess whether the duration detected from each instrumented segment also represented the broader movement bout, rather than only the movement of that segment. For the sacral placement, local and common references coincided, as both were based on the S1 marker. The common reference was applied only to whole-body tasks, whereas for isolated upper-limb tasks only the local reference was considered, since movement of the instrumented arm was the primary outcome of interest. A representative example of the detection procedure for both systems is shown in Figure 3.

### 2.4. Statistical Analysis

Statistical analyses were performed in MATLAB (MathWorks, Natick, MA, USA). For the main agreement analyses, repeated-trial durations were averaged within each participant, placement, task, and reference definition; a complementary trial-level analysis was also performed. As data were not normally distributed (Shapiro–Wilk test, *p* < 0.05), descriptive statistics are reported as median (interquartile range, IQR).

The association between IMU- and MoCap-derived bout durations was assessed using Spearman’s rank correlation coefficient (ρ). Agreement was evaluated using the intraclass correlation coefficient (ICC(2,1)), selected because absolute agreement, rather than consistency, was of interest, and Bland–Altman analysis. Bias and 95% limits of agreement (LoA) were calculated from participant-level paired differences. Ninety-five percent confidence intervals (95% CIs) were calculated for ICC estimates. As the paired differences deviated from normality (Shapiro–Wilk test, *p* < 0.05) for some task–placement combinations, 95% CIs for the LoA were estimated by participant-level bootstrap resampling (5000 iterations).

Error was quantified using the mean absolute percentage error (MAPE), calculated as:

(2) $$MAPE=\;\frac{1}{n}\sum_{i=1}^{n}\left|\;\frac{{MoCap}_{i}-{IMU}_{i}}{{MoCap}_{i}}\right|\times 100$$

where IMU_i_ and MoCap_i_ are the participant-level mean IMU- and MoCap-derived durations and n is the number of participants. Its 95% CIs were estimated with the same bootstrap procedure.

Differences among placements were assessed using the Friedman test on participant-level MAPE, separately for each whole-body task and reference definition. Only participants with complete data across all seven placements were included in each comparison; the number of participants without valid data for each placement, task, and reference definition is reported in Table S1. Significant Friedman tests were followed by Wilcoxon signed-rank tests with Holm adjustment.

As a sensitivity analysis, a trial-level analysis using generalized estimating equations (GEE) was performed to evaluate whether placement-related differences were maintained when repeated trials were retained rather than averaged within participants. The GEE analysis used all valid trials, including those from participants excluded from the Friedman comparisons. For each trial, the absolute percentage error (APE) was calculated as (Equation (3)):

(3) $$APE=\;\left|\;\frac{{MoCap}_{ij}-{IMU}_{ij}}{{MoCap}_{ij}}\right|\times 100$$

where IMU_ij_ and MoCap_ij_ are the IMU- and MoCap-derived durations of trial j of participant i. To reduce the skewness of the error distribution, APE was log-transformed as:

(4) logAPE= ln(1+APE)

The transformed error was used as the dependent variable, with sensor placement as the predictor, participant as clustering variable, and an exchangeable correlation structure. Separate models were fitted for each task and reference definition; for upper-limb tasks, the dominant and non-dominant wrists were compared.

To provide a descriptive synthesis of placement performance, a composite ranking was derived from three agreement indicators: ICC, MAPE, and LoA width. For each whole-body task, the seven placements were ranked from 1 to 7 on each indicator independently. Rank 1 corresponded to the highest ICC, the lowest MAPE, and the narrowest LoA width. Indicator-specific ranks were averaged to obtain a task-specific composite score, and task-specific scores were subsequently averaged to yield an overall placement score. The procedure was applied separately to the local and common references. The common reference served as the primary criterion, and the local reference was used to support the interpretation of segment-specific performance. No composite ranking was computed for upper-limb tasks, which were evaluated against the local reference only.

The composite ranking was regarded as descriptive and was not interpreted as evidence of statistically significant differences between placements. Its stability was quantified by repeating the entire ranking procedure on 2000 participant-level bootstrap samples. The proportion of resamples in which each placement ranked among the three highest positions was used as the stability index.

## 3. Results

### 3.1. Agreement with the Local Reference

Agreement with the local reference varied across tasks and placements (Table 1). The dominant ankle showed consistently high agreement, with ICC values of 0.821, 0.873, and 0.993 for walking, TUG, and Step Test, respectively, and corresponding MAPE values of 5.78%, 3.72%, and 1.51%. During walking, the sacral placement showed poor agreement (ICC = 0.104; MAPE = 21.94%), with a systematic underestimation of bout duration (bias = −0.96 s). The dominant foot also showed relatively high error (MAPE = 18.33%). In the TUG, the dominant foot and ankle showed the highest local agreement (ICC = 0.911 and 0.873; MAPE = 3.45% and 3.72%, respectively), whereas the dominant wrist showed substantially poorer agreement (ρ = 0.153; ICC = 0.050; MAPE = 27.63%). The non-dominant wrist showed lower error than the dominant wrist (MAPE = 8.63% vs. 27.63%). During the Step Test, agreement was generally high. The dominant ankle showed the highest agreement (ICC = 0.993; MAPE = 1.51%), while the dominant wrist showed the largest error (MAPE = 12.03%) despite a relatively high correlation (ρ = 0.939).

For the upper-limb tasks, both wrist placements showed high agreement with the local reference (Table 2). During reaching, the non-dominant and dominant wrists achieved ICC values of 0.896 and 0.879, with corresponding MAPE values of 6.35% and 7.07%. Agreement was higher during the hand-to-mouth task, particularly for the non-dominant wrist (ICC = 0.994; MAPE = 1.75%), although the dominant wrist also showed high agreement (ICC = 0.967; MAPE = 2.48%). Overall, differences between wrists were relatively small during isolated upper-limb movements, in contrast to the more pronounced differences observed during the TUG and Step Test.

The distribution of the participant-level paired IMU-MoCap differences showed significant departures from normality for some task-placement combinations. Therefore, uncertainty around the Bland–Altman limits of agreement was quantified using participant-level bootstrap 95% CIs.

### 3.2. Agreement with the Common Reference

Using the S1 marker as a common reference changed agreement most markedly in the TUG (Table 3). The dominant ankle and foot showed substantial reductions in ICC compared with the local reference (0.873 to 0.096 and 0.911 to 0.178, respectively), with MAPE values increasing to 19.67% and 20.30%. Their IMU-derived durations were shorter than the common reference (7.15 and 7.26 s vs. 8.82 s), with biases of −1.76 and −1.85 s. In contrast, the non-dominant wrist showed the lowest error among the non-sacral placements in the TUG (MAPE = 8.67%), while the sacrum showed the lowest error overall (MAPE = 6.43%), noting that for this placement the local and common references coincided by construction. Local-common differences were smaller in the Step Test, where most placements showed high agreement with the common reference.

### 3.3. Trial-Level Sensitivity Analysis

The complementary trial-level GEE analysis confirmed a significant effect of sensor placement for all whole-body tasks. Against the local reference, placement significantly affected trial-level error during walking (Wald χ^2^(6) = 174.71, *p* < 0.001), TUG (Wald χ^2^(6) = 133.39, *p* < 0.001), and Step Test (Wald χ^2^(6) = 138.33, *p* < 0.001). Similar results were obtained against the common reference for walking (Wald χ^2^(6) = 34.33, *p* < 0.001), TUG (Wald χ^2^(6) = 79.61, *p* < 0.001), and Step Test (Wald χ^2^(6) = 104.08, *p* < 0.001).

For the isolated upper-limb tasks, no significant difference in trial-level error was found between dominant and non-dominant wrist placements during reaching (Wald χ^2^(1) = 2.71, *p* = 0.100) or hand-to-mouth movements (Wald χ^2^(1) = 0.85, *p* = 0.358). Overall, the trial-level analyses were consistent with the participant-level findings and indicated that the observed placement-related differences were not solely attributable to averaging repeated trials within participants or to the exclusion of participants with incomplete data from the complete-case comparisons.

### 3.4. Differences Among Placements and Overall Ranking

Placement had a significant effect on measurement error in all three whole-body tasks, both against the local reference (Friedman test on participant-level MAPE: walking χ^2^(6) = 47.96, *p* < 0.001, n = 24; TUG χ^2^(6) = 47.04, *p* < 0.001, n = 24; Step Test χ^2^(6) = 52.64, *p* < 0.001, n = 23) and against the common reference (walking χ^2^(6) = 29.71, *p* < 0.001, n = 24; TUG χ^2^(6) = 43.41, *p* < 0.001, n = 25; Step Test χ^2^(6) = 47.20, *p* < 0.001, n = 23). The different sample sizes reflected the complete-case requirement of the Friedman test, which resulted in the exclusion of 3 participants for walking, 3 (local reference) and 2 (common reference) for TUG, and 4 for the Step Test (Table S1). Missing data were due to acquisition issues, including unavailable IMU recordings and unavailable reference marker trajectories, and were not related to failed movement detection or segmentation.

Holm-adjusted post hoc comparisons indicated that differences between individual placements were task-dependent. In particular, against the common reference, the non-dominant wrist showed lower error than the dominant wrist during the TUG (adjusted *p* = 0.0086) and Step Test (adjusted *p* = 0.0007), whereas no significant difference between wrist placements was observed during walking.

**Figure 4:**
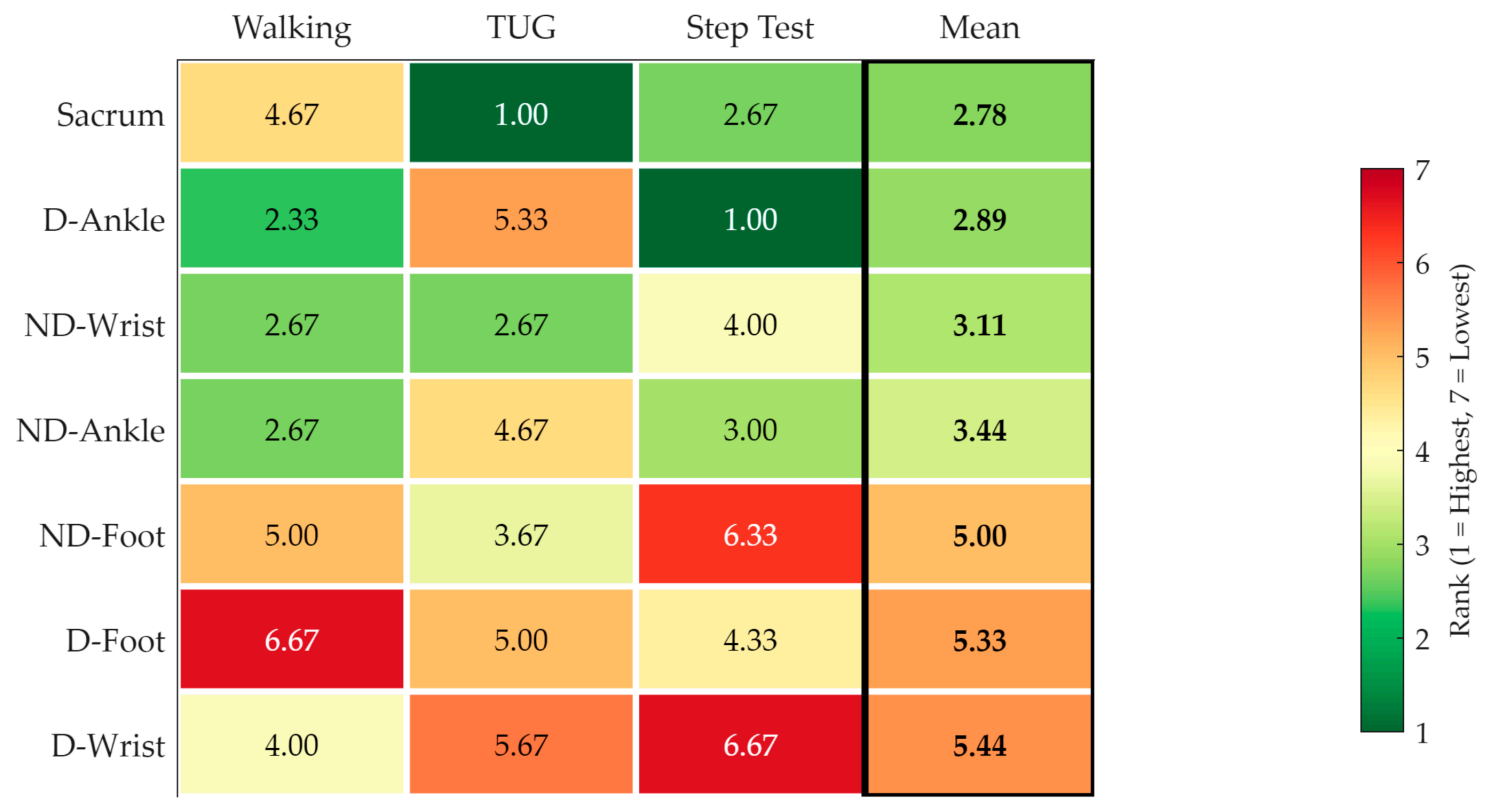
Ranking of sensor placements across the three whole-body tasks using the common reference. Lower ranks indicate better performance.

Figure 4 shows the descriptive composite ranking against the common reference.

The sacrum obtained the most favourable overall rank (2.78), closely followed by the dominant ankle (2.89) and the non-dominant wrist (3.11). Both wrists showed high agreement during upper-limb tasks (ICC = 0.879–0.994).

Bootstrap analysis showed that the exact ordering of the highest-ranked placements was not completely stable, although the same placements tended to remain within the most favourable group. The sacrum appeared among the three highest-ranked placements in 94.4% of bootstrap samples, the non-dominant wrist in 76.3%, and the dominant ankle in 76.0%. These results support interpretation of the composite ranking as a descriptive synthesis rather than as evidence of a strict hierarchy among placements.

To assess the potential influence of the inherent correspondence between the sacral IMU placement and the S1 common reference, the composite ranking was recalculated after excluding the sacral placement. The non-dominant wrist (2.33) and dominant ankle (2.44) obtained the two most favorable scores, followed by the non-dominant ankle (2.78). In the corresponding bootstrap analysis, these three placements appeared among the three highest-ranked locations in 98.4%, 94.5%, and 90.9% of resamples, respectively.

Because it combined acceptable whole-body performance with accurate upper-limb bout duration estimation, the non-dominant wrist is proposed as a promising candidate for further evaluation in subsequent free-living monitoring.

## 4. Discussion

This study evaluated seven IMU placements for the temporal quantification of predefined active movement bouts against a concurrent optoelectronic MoCap reference. Performance varied according to the placement site, motor task, and reference definition. The sacrum and dominant ankle occupied the leading positions in the descriptive composite ranking against the common reference, although performance was not uniform across tasks. Placement-related differences were confirmed by the trial-level analysis, which retained variability across repeated trials. More importantly, the local-common comparison showed that accurate estimation of segment-specific movement duration does not necessarily translate into equally accurate representation of the broader movement bout.

The dominant ankle showed consistently high agreement with the local reference, with ICC values of 0.821, 0.873, and 0.993 for walking, TUG, and Step Test, respectively. Its favourable performance during walking and the Step Test is consistent with the established use of distal lower-limb sensors for locomotor assessment [27]. The TUG, however, revealed a marked dissociation between segment-specific and broader movement duration. For the dominant ankle and foot, ICC decreased from 0.873 and 0.911 against the local reference to 0.096 and 0.178 against the common reference, respectively. Their IMU-derived durations were also shorter than the S1-based reference, with biases of −1.76 and −1.85 s. This pattern may reflect the composite structure of the TUG, which combines standing up, walking, turning, and sitting down [36]. Participants may adopt different trunk inclinations and lower-limb movements during sit-to-stand and stand-to-sit, while turning may be performed with different step numbers or body segment coordination.

Previous work has shown that inertial signals from different body segments are not always temporally aligned during TUG transitions, and that the agreement of IMU-derived durations with motion capture varies across subtasks [37,38]. These findings provide a plausible explanation for the greater local-common differences observed in the TUG than in the more continuous Step Test. However, phase-specific analyses would be needed to identify the precise source of the discrepancy. The present results therefore suggest that accurate tracking of a segment does not necessarily ensure accurate representation of a broader movement bout, highlighting the importance of evaluating wearable-derived outcomes against a reference appropriate to the intended measurement [23].

The sacral results should be interpreted in light of the reference definition. The S1 marker was used as a common proxy for the comparison of whole-body movement duration across sensor placements, rather than as an absolute measure of whole-body movement. The sacrum obtained the most favorable descriptive composite rank against the common reference (2.78), closely followed by the dominant ankle (2.89). For the sacral placement, local and common references coincide with construction, which should be considered when interpreting its position in the ranking. However, when the ranking was recalculated excluding the sacral placement, the non-dominant wrist and dominant ankle remained the two most favorable configurations, indicating that the relative ranking of the other placements did not depend on this correspondence. Bootstrap analysis further showed that the same placements consistently formed the leading group, with the sacrum, non-dominant wrist, and dominant ankle appearing among the three highest-ranked placements in 94.4%, 76.3%, and 76.0% of resamples, respectively, whereas their exact ordering varied across resamples.

Nevertheless, performance remained task-dependent, with the sacrum showing lower agreement during walking (ICC = 0.104; MAPE = 21.94%) than during the TUG (ICC = 0.715; MAPE = 6.43%) and Step Test (ICC = 0.973; MAPE = 3.76%), with a systematic underestimation of bout duration during walking (bias = −0.96 s).

This underestimation is consistent with the lower acceleration amplitude at the sacrum during gait initiation and termination, where trunk acceleration rises and decays more gradually than at distal segments. With a fixed onset/offset threshold, this is expected to truncate the detected bout at both ends, producing the observed systematic negative bias. These findings indicate that the favourable overall ranking does not imply uniformly high agreement across movement tasks.

The wrist results further support a task-dependent interpretation. During whole-body tasks, the non-dominant wrist showed lower error than the dominant wrist, particularly in the TUG and Step Test (MAPE against the common reference: 8.67% and 4.16% vs. 18.40% and 12.52%). These differences were significant in Holm-adjusted post hoc comparisons, whereas no significant difference between wrists was observed during walking. Differences in raw acceleration output and activity estimates between dominant- and non-dominant-wrist accelerometry have been previously reported [22,39]. During isolated upper-limb tasks, by contrast, both wrists showed high local agreement (ICC = 0.879–0.994), with no significant difference between sides at the trial level. This is consistent with previous work demonstrating the feasibility of wrist-worn accelerometers for monitoring elementary upper-limb movements, including reaching and bringing a cup to the mouth [40]. Together, these results support the potential of wrist placement for monitoring both whole-body and upper-limb movement, while highlighting the importance of the specific task and outcome considered.

Taken together, these findings suggest that sensor placement should be selected according to the intended monitoring outcomes. For free-living applications, where a single device may be required to capture different types of movement, the most suitable placement is not necessarily the one showing the highest agreement in an individual task, but one providing a reasonable balance of performance across movement domains. The sacrum and the dominant ankle occupied the first two positions in the common-reference ranking. The non-dominant wrist ranked third, obtaining the most favorable score once the sacral placement was excluded, and combined acceptable whole-body performance with accurate upper-limb duration estimation. Given the descriptive nature of the ranking, however, these positions should not be read as statistically distinct levels of performance. The non-dominant wrist is therefore best regarded as a promising candidate for further evaluation in a single-sensor configuration, rather than as an optimal placement. Previous large-scale studies have demonstrated the use of wrist-worn accelerometers for continuous physical activity monitoring and the characterization of different activity and sleep behaviours [41,42]. Its performance will need to be confirmed under the more heterogeneous movement conditions of the subsequent free-living phase.

Beyond technical performance, sensor placement should also be considered in relation to the practical requirements of prolonged free-living monitoring, including overnight wear. Comfort, acceptability, and interference with daily activities or sleep may influence adherence, particularly in populations with sensory sensitivities. Continuous wrist-worn monitoring has demonstrated feasibility in large population studies [42], while the acceptability of wrist- and ankle-worn devices has also been examined in children and adolescents with ASD [43]. These findings support considering a wrist-worn configuration for prolonged monitoring, although individual tolerability remains an important consideration. Since wearability was not directly assessed in the present study, the relative acceptability of the different placements remains to be established.

Several limitations should be considered. First, the sample was small and homogeneous, consisting of young, healthy, right-handed adults, and the tasks were performed under controlled laboratory conditions. The findings, including the relative performance of the dominant and non-dominant wrists, may therefore not generalize to older adults, clinical populations, or left-handed individuals. Second, the study was designed as a controlled comparison of sensor placements based on the duration of predefined motor tasks, not as a validation of a free-living event-detection algorithm. Event-level metrics such as sensitivity, precision, false-positive detections, or onset/offset timing errors were therefore outside its scope, and the results should not be interpreted as evidence of detection accuracy. Agreement was primarily assessed at the participant level, although the complementary trial-level analysis, which retained within-participant variability across available valid trials, yielded consistent findings. Third, the segmentation parameters were chosen to capture the complete predefined sequence within each trial, with short gaps merged to avoid fragmenting consecutive repetitions. Their suitability for continuous free-living recordings, where bouts occur spontaneously and with greater heterogeneity, remains to be established. Finally, the composite ranking is a descriptive synthesis of ICC, MAPE, and LoA width. Although the group of leading placements was reasonably stable across bootstrap resamples, their exact ordering varied, and the ranking should not be interpreted as evidence of statistical superiority between placements.

These findings provide a technical basis for selecting a sensor configuration and guiding the subsequent free-living monitoring phase. Further work will focus on adapting the processing approach to more heterogeneous movement conditions, developing task-specific outcomes where appropriate, and evaluating the feasibility of prolonged device use.

## 5. Conclusions

This preliminary study provides a technical basis for comparing wearable sensor placements for movement bout duration estimation during controlled, predefined motor tasks. The findings highlight the importance of considering the intended monitoring outcome when selecting a sensor location. Although the sacrum and dominant ankle occupied the leading positions in the descriptive composite ranking against the common reference, the non-dominant wrist showed a favourable combination of performance across whole-body and upper-limb tasks, and obtained the most favorable score when the sacral placement was excluded. These placement-related findings were confirmed by trial-level analyses accounting for variability across repeated trials. Given the descriptive nature of the ranking and the young, healthy, right-handed sample, the present findings do not establish an optimal sensor location. Rather, they identify the non-dominant wrist as a promising candidate for further evaluation as a single-sensor configuration, where a reasonable balance across different types of movement is particularly relevant.

Future work will investigate its performance under more heterogeneous movement conditions and will specifically address free-living bout detection performance, together with the feasibility of prolonged monitoring.

## Figures and Tables

**Table 1:** Agreement between IMU-derived and MoCap-derived bout durations using the local reference for the whole-body tasks.

Task	Placement	IMU (s)	MoCap (s)	ρ	ICC (95% CI)	Bias (s)	Lower LoA (95% CI), s	Upper LoA (95% CI), s	MAPE, % (95% CI)
Walking	D-Ankle	4.29 (3.24–6.12)	4.40 (3.51–6.21)	0.927	0.821 (0.649–0.904)	−0.20	−0.76 (−0.924 to −0.539)	0.37 (0.111 to 0.632)	5.78 (4.17–7.50)
	ND-Ankle	4.20 (3.15–5.79)	4.35 (3.52–6.04)	0.804	0.486 (0.222–0.637)	−0.41	−1.34 (−1.617 to −1.013)	0.52 (0.154 to 0.815)	11.07 (8.77–13.72)
	D-Wrist	4.53 (3.76–5.45)	4.39 (3.44–6.17)	0.765	0.503 (0.152–0.706)	−0.10	−1.21 (−1.590 to −0.817)	1.00 (0.643 to 1.287)	9.59 (7.56–11.94)
	ND-Foot	4.26 (3.16–6.08)	4.35 (3.52–6.04)	0.803	0.439 (0.079–0.706)	−0.16	−1.49 (−1.897 to −0.991)	1.17 (0.385 to 1.939)	11.09 (7.73–15.17)
	ND-Wrist	4.25 (3.65–5.05)	4.39 (3.52–6.15)	0.622	0.399 (0.132–0.677)	−0.45	−1.58 (−1.973 to −1.078)	0.68 (0.396 to 0.874)	10.71 (7.53–14.35)
	Sacrum	3.28 (2.95–3.64)	4.34 (3.67–5.97)	0.464	0.104 (−0.007–0.209)	−0.96	−1.80 (−2.077 to −1.438)	−0.13 (−0.389 to 0.077)	21.94 (18.95–24.98)
	D-Foot	4.93 (3.37–7.08)	4.40 (3.49–6.20)	0.714	0.111 (−0.121–0.524)	+0.46	−2.39 (−2.856 to −1.458)	3.31 (1.477 to 4.685)	18.33 (8.48–29.89)
TUG	D-Foot	7.26 (6.60–7.77)	7.17 (6.49–7.65)	0.860	0.911 (0.789–0.967)	+0.11	−0.50 (−0.639 to −0.317)	0.71 (0.399 to 0.993)	3.45 (2.30–4.90)
	D-Ankle	7.15 (6.58–7.74)	7.17 (6.49–7.65)	0.841	0.873 (0.735–0.948)	+0.12	−0.58 (−0.767 to −0.352)	0.81 (0.513 to 1.071)	3.72 (2.42–5.17)
	ND-Ankle	7.95 (7.05–8.60)	7.82 (6.86–8.49)	0.844	0.773 (0.535–0.955)	+0.15	−1.02 (−1.503 to −0.344)	1.32 (0.533 to 2.112)	4.34 (2.44–7.20)
	Sacrum	9.16 (8.41–9.86)	8.82 (8.19–9.51)	0.719	0.715 (0.465–0.849)	+0.18	−1.15 (−1.374 to −0.843)	1.52 (1.003 to 1.926)	6.43 (4.59–8.40)
	ND-Foot	8.21 (7.31–8.83)	7.82 (6.86–8.49)	0.851	0.691 (0.484–0.896)	+0.49	−0.77 (−1.209 to −0.193)	1.74 (0.838 to 2.587)	6.44 (3.81–9.86)
	ND-Wrist	9.08 (8.36–9.90)	8.66 (8.13–9.33)	0.524	0.475 (0.068–0.867)	+0.44	−1.74 (−2.228 to −0.860)	2.63 (1.091 to 3.828)	8.63 (4.78–13.64)
	D-Wrist	10.38 (9.14–11.20)	8.28 (7.51–9.01)	0.153	0.050 (−0.076–0.205)	+1.95	−1.41 (−2.153 to −0.311)	5.31 (4.297 to 6.056)	27.63 (20.38–35.41)
Step Test	D-Ankle	21.16 (18.58–23.99)	21.28 (18.64–24.52)	0.977	0.993 (0.986–0.997)	−0.25	−0.89 (−1.164 to −0.604)	0.38 (0.080 to 0.618)	1.51 (1.07–2.06)
	Sacrum	21.80 (18.88–24.77)	21.06 (17.80–24.36)	0.983	0.973 (0.950–0.984)	+0.73	−0.05 (−0.237 to 0.193)	1.52 (1.181 to 1.836)	3.76 (2.89–4.74)
	ND-Ankle	22.10 (18.70–24.77)	22.02 (18.82–24.91)	0.950	0.983 (0.948–0.996)	−0.13	−1.47 (−2.009 to −0.743)	1.20 (0.261 to 2.157)	1.93 (1.18–3.05)
	D-Foot	21.33 (18.32–24.62)	21.33 (18.64–25.12)	0.971	0.967 (0.903–0.997)	−0.35	−2.26 (−3.756 to −0.673)	1.56 (0.283 to 2.420)	2.38 (1.06–4.59)
	ND-Wrist	21.86 (18.75–24.51)	21.14 (17.89–24.44)	0.973	0.972 (0.946–0.985)	+0.67	−0.45 (−0.643 to −0.195)	1.78 (1.320 to 2.147)	3.50 (2.34–4.74)
	ND-Foot	23.05 (19.83–26.06)	22.02 (18.82–24.91)	0.913	0.908 (0.809–0.960)	+1.07	−1.55 (−2.006 to −0.957)	3.68 (2.479 to 4.657)	5.60 (3.51–7.94)
	D-Wrist	24.43 (20.66–27.44)	21.13 (17.90–24.45)	0.939	0.791 (0.653–0.869)	+2.57	−0.07 (−0.782 to 0.860)	5.21 (4.248 to 6.014)	12.03 (9.66–14.53)

D: dominant; ND: non-dominant. Durations are median (IQR). ρ: Spearman’s rank correlation coefficient; ICC: absolute-agreement intraclass correlation coefficient; LoA: 95% limits of agreement; Bias was calculated as IMU − MoCap; positive values indicate overestimation by the IMU, whereas negative values indicate underestimation. LoA represents the range within which approximately 95% of individual differences between the two methods are expected to fall. Values in parentheses following ICC and MAPE indicate 95% confidence intervals. For lower and upper LoA, the first value represents the LoA estimate and the subsequent parenthetical range represents its bootstrap 95% CI.

**Table 2:** Agreement between IMU-derived and MoCap-derived bout durations using the local reference for the upper-limb tasks.

Task	Placement	IMU (s)	MoCap (s)	ρ	ICC (95% CI)	Bias (s)	Lower LoA (95% CI), s	Upper LoA (95% CI), s	MAPE, % (95% CI)
Reaching	ND-Wrist	13.38 (11.71–14.82)	12.09 (11.10–14.07)	0.892	0.896 (0.790–0.948)	+0.60	−1.06 (−1.467 to −0.514)	2.27 (1.489 to 2.921)	6.35 (4.70–8.22)
	D-Wrist	13.23 (11.48–14.72)	11.99 (10.95–13.75)	0.867	0.879 (0.767–0.936)	+0.72	−0.98 (−1.406 to −0.431)	2.41 (1.647 to 3.038)	7.07 (5.18–9.16)
Hand-to-Mouth	ND-Wrist	11.62 (10.11–13.04)	11.80 (10.27–13.39)	0.998	0.994 (0.989–0.996)	−0.20	−0.38 (−0.459 to −0.270)	−0.02 (−0.073 to 0.015)	1.75 (1.41–2.15)
	D-Wrist	12.04 (10.05–13.25)	12.55 (10.14–13.47)	0.969	0.967 (0.902–0.995)	−0.29	−1.12 (−1.684 to −0.391)	0.53 (0.032 to 0.777)	2.48 (1.57–3.76)

D: dominant; ND: non-dominant. Durations are median (IQR). Abbreviations as in Table 1.

**Table 3:** Agreement between IMU-derived bout durations and the common reference for the three whole-body tasks.

Task	Placement	IMU (s)	MoCap (s)	ρ	ICC (95% CI)	Bias (s)	Lower LoA (95% CI), s	Upper LoA (95% CI), s	MAPE, % (95% CI)
Walking	D-Ankle	4.29 (3.24–6.12)	4.34 (3.67–5.97)	0.883	0.563 (0.184–0.794)	−0.12	−1.09 (−1.430 to −0.682)	0.85 (0.468 to 1.176)	7.25 (4.64–10.04)
	ND-Ankle	4.20 (3.15–5.79)		0.862	0.549 (0.303–0.690)	−0.37	−1.21 (−1.477 to −0.912)	0.47 (0.205 to 0.673)	9.12 (6.32–12.08)
	D-Foot	4.93 (3.37–7.08)		0.684	0.018 (−0.175–0.318)	+0.51	−2.45 (−2.978 to −1.608)	3.48 (1.785 to 4.790)	21.13 (11.64–32.67)
	ND-Foot	4.26 (3.16–6.08)		0.867	0.492 (0.214–0.713)	−0.13	−1.39 (−1.762 to −0.899)	1.12 (0.371 to 1.841)	9.97 (6.71–13.91)
	Sacrum	3.28 (2.95–3.64)		0.464	0.104 (−0.006–0.215)	−0.96	−1.80 (−2.076 to −1.449)	−0.13 (−0.384 to 0.066)	21.94 (18.95–25.03)
	ND-Wrist	4.25 (3.65–5.05)		0.653	0.584 (0.352–0.731)	−0.34	−1.22 (−1.430 to −0.944)	0.53 (0.258 to 0.732)	9.54 (7.03–12.31)
	D-Wrist	4.53 (3.76–5.45)		0.755	0.451 (−0.012–0.729)	−0.07	−1.23 (−1.698 to −0.740)	1.08 (0.693 to 1.384)	9.06 (6.47–11.81)
TUG	D-Ankle	7.15 (6.58–7.74)	8.82 (8.19–9.51)	0.313	0.096 (−0.013–0.188)	−1.76	−3.52 (−3.891 to −3.000)	0.00 (−0.685 to 0.523)	19.67 (16.24–22.95)
	ND-Ankle	7.95 (7.05–8.60)		0.395	0.291 (0.084–0.445)	−1.03	−3.01 (−3.374 to −2.525)	0.96 (0.207 to 1.577)	13.36 (10.53–16.19)
	D-Foot	7.26 (6.60–7.77)		0.564	0.178 (0.072–0.256)	−1.85	−3.32 (−3.626 to −2.905)	−0.39 (−0.835 to −0.074)	20.30 (17.55–23.11)
	ND-Foot	8.21 (7.31–8.83)		0.491	0.422 (0.130–0.610)	−0.71	−2.60 (−2.953 to −2.110)	1.19 (0.506 to 1.658)	11.07 (8.62–13.51)
	Sacrum	9.16 (8.41–9.86)		0.719	0.715 (0.476–0.848)	+0.18	−1.15 (−1.370 to −0.843)	1.52 (0.988 to 1.916)	6.43 (4.55–8.45)
	ND-Wrist	9.08 (8.36–9.90)		0.570	0.556 (0.271–0.773)	+0.33	−1.55 (−1.831 to −1.085)	2.21 (1.412 to 2.800)	8.67 (5.98–11.59)
	D-Wrist	10.38 (9.14–11.20)		0.425	0.201 (0.037–0.381)	+1.40	−1.26 (−1.822 to −0.429)	4.06 (3.201 to 4.720)	18.40 (13.62–23.26)
Step Test	D-Ankle	21.16 (18.58–23.99)	21.06 (17.80–24.36)	0.970	0.985 (0.970–0.992)	+0.46	−0.32 (−0.703 to 0.041)	1.24 (0.893 to 1.525)	2.62 (2.05–3.25)
	ND-Ankle	22.10 (18.70–24.77)		0.942	0.968 (0.917–0.988)	+0.66	−0.63 (−1.138 to 0.123)	1.96 (1.105 to 2.807)	3.56 (2.71–4.79)
	D-Foot	21.33 (18.32–24.62)		0.967	0.962 (0.906–0.990)	+0.39	−1.61 (−3.226 to 0.128)	2.40 (0.957 to 3.336)	3.74 (2.44–5.74)
	ND-Foot	23.05 (19.83–26.06)		0.910	0.837 (0.711–0.909)	+1.87	−0.87 (−1.334 to −0.250)	4.61 (3.342 to 5.502)	9.24 (6.90–11.85)
	Sacrum	21.80 (18.88–24.77)		0.983	0.973 (0.950–0.984)	+0.73	−0.05 (−0.231 to 0.198)	1.52 (1.178 to 1.837)	3.76 (2.87–4.73)
	ND-Wrist	21.86 (18.75–24.51)		0.969	0.966 (0.942–0.978)	+0.82	−0.12 (−0.332 to 0.159)	1.77 (1.463 to 1.990)	4.16 (3.12–5.25)
	D-Wrist	24.43 (20.66–27.44)		0.936	0.779 (0.638–0.861)	+2.66	−0.01 (−0.777 to 0.963)	5.33 (4.353 to 6.159)	12.52 (10.11–14.94)

D: dominant; ND: non-dominant. Durations are median (IQR). Abbreviations as in Table 1.

## Data Availability

Data are available from the corresponding author upon reasonable request.

## Supplementary Materials

The following supporting information can be downloaded at: https://www.mdpi.com/article/10.3390/bioengineering13101206/s1, Table S1: Number of participants without valid IMU–MoCap paired data for each sensor placement, task and reference definition.

## Abbreviations

The following abbreviations are used in this manuscript:

IMU	Inertial Measurement Unit
MoCap	Motion Capture
D-	Dominant
ND-	Non-Dominant
ENMO	Euclidean Norm Minus One
TUG	Timed Up and Go
ICC	Intraclass Correlation Coefficient
LoA	Limits of Agreement
MAPE	Mean Absolute Percentage Error
CI	Confidence Interval
IQR	Interquartile Range
MAD	Median Absolute Deviation
GEE	Generalized Estimating Equations
COM	Center of Mass
