# Supplementary material for: Evaluation of Wearable Sensor Placement for Movement Bout Duration Estimation: A Comparative Feasibility Study
Source: Bioengineering (Basel). 2026 Oct 16;13(10):1206. doi: 10.3390/bioengineering13101206 (PMC13701408; doi:10.3390/bioengineering13101206)
Supplement: Supplementary file 1 [file bioengineering-13-01206-s001.zip › bioengineering-4618173-supplementary.pdf]

Supplementary Materials

Table S1. Number of participants (out of 27) without valid IMU–MoCap paired data for each sensor placement, task and reference definition.

Placement	Walking		TUG		Step Test		Reaching	Hand-to-Mouth
	**Local**	**Common**	**Local**	**Common**	**Local**	**Common**	**Local**	**Local**
D-Ankle	2	2	1	1	2	2	n.a.	n.a.
ND-Ankle	1	2	0	0	1	1	n.a.	n.a.
D-Foot	3	3	1	1	2	2	n.a.	n.a.
ND-Foot	1	2	0	0	1	1	n.a.	n.a.
D-Wrist	1	2	1	0	2	2	1	1
ND-Wrist	1	2	1	0	1	1	1	1
Sacrum	2	2	0	0	1	1	n.a.	n.a.
**Participants excluded, n**	**3**	**3**	**3**	**2**	**4**	**4**	**1**	**1**
**Participants analysed, n**	**24**	**24**	**24**	**25**	**23**	**23**	**26**	**26**

D: dominant; ND: non-dominant; n.a.: not applicable. The common reference was not applied to the upper-limb tasks. Participants excluded: participants with missing data for at least one placement, who were therefore excluded from the complete-case comparisons (Friedman tests for whole-body tasks; wrist comparison for upper-limb tasks).
